# How to Evaluate the Efficacy of Manipulations in Spine Disorders—A Comprehensive Review of New and Traditional Outcome Measures

**DOI:** 10.3390/clinpract14040119

**Published:** 2024-07-29

**Authors:** Giacomo Farì, Carlo Mariconda, Laura Dell’Anna, Francesco Quarta, Danilo Donati, Cristiano Sconza, Vincenzo Ricci, Giustino Varrassi, Valeria Coco, Alessandro Manelli, Ennio Spadini, Maria Teresa Giglio, Andrea Bernetti

**Affiliations:** 1 National Council of the Manual Medicine Section, Italian Society of Physical and Rehabilitation Medicine (SIMFER), 00198 Rome, Italy; carlo.mariconda@gradenigo.it (C.M.); valecoco@yahoo.it (V.C.); emanelli@libero.it (A.M.); ennio.spadini@gmail.com (E.S.); 2 Department of Experimental Medicine (Di.Me.S), University of Salento, 73100 Lecce, Italy; andrea.bernetti@unisalento.it; 3 Department of Rehabilitation Sciences, Humanitas Gradenigo Hospital, 10153 Turin, Italy; 4 Department of Translational Biomedicine and Neuroscience (DiBraiN), Aldo Moro University, 70121 Bari, Italy; lauradellanna@gmail.com; 5 Department of Biological and Environmental Science and Technologies (Di.S.Te.B.A.), University of Salento, 73100 Lecce, Italy; francesco.quarta6@studenti.unisalento.it; 6 Clinical and Experimental Medicine, University of Modena and Reggio Emilia, 41121 Modena, Italy; danilo.donati@unimore.it; 7 IRCCS Humanitas Research Hospital, 20089 Rozzano, Italy; cristiano.sconza@gmail.com; 8 Physical and Rehabilitation Medicine Unit, Luigi Sacco University Hospital, 20157 Milano, Italy; ricci.vincenzo@asst-fbf-sacco.it; 9 Paolo Procacci Foundation (PPF), 00193 Rome, Italy; g.varrassi@fondazioneprocacci.org; 10 Anaesthesia and Intensive Care Unit, Policlinico Hospital, 70124 Bari, Italy; mariateresa.giglio@uniba.it

**Keywords:** manual therapy, spine pain, outcome, measure

## Abstract

Spine pain (SP) is the most common musculoskeletal disorder that causes transitional forms of motor disability. Considering its affordability and safety, manipulative therapy (MT) stands as one of the primary therapeutic approaches for SP and the related dysfunctional consequences. However, it is still difficult to assess and quantify the results of this treatment since there is a lack of objective evaluation tools in the available scientific literature. Thus, the purpose of this comprehensive review is to summarize the main outcomes used to evaluate the effectiveness of spine manipulations, focusing on their strengths and weaknesses. An extensive review of the PubMed, Cochrane, and Embase databases was performed to identify the literature of the last ten years regarding MT and the related assessment tools. A total of 12 studies met the inclusion criteria. The analyzed literature indicates that a wide range of outcome measures have been used to assess the effectiveness of spine MT. Pain is the main aspect to be investigated but it remains difficult to elucidate since it is strongly linked to various dimensions such as self-perception and psychological aspects. Therefore, it seems necessary to include new tools for evaluating the effects of spine MT, with the aim of exploiting new technologies and taking into consideration the SP biomechanical and biopsychosocial aspects.

## 1. Introduction

Spine pain (SP) is the most common musculoskeletal disorder and imposes a significant public health burden globally [1]. In fact, neck pain (NP) and low back pain (LBP) represent a great cause of motor disability and imply reduced job productivity and high healthcare spending [2,3]. SP is defined as a musculoskeletal complaint with nociceptive, neuropathic, or nociplastic characteristics. It refers to one or more anatomical districts of the spine, from the cervical segment to the coccyx, and can radiate to the limbs. SP is not a single condition but rather a symptom and, in the vast majority of cases, it is considered as non-specific since it cannot be attributed to a specific osteoarticular or myotendinous disease and must be considered from the perspective of a biopsychosocial model. This model outlines the complexity of pain considering biophysical, psychological, social, and genetic aspects and comorbidities [4]. Several risk factors are related to SP such as demographic, health, occupational, psychologic, and spinal anatomy factors [5]. Moreover, the classification of SP is shape-shifting and fragmented, which makes it a difficult task [6]. The challenge of SP management and treatment is therefore always current and involves many important medicine sectors. Among one of the most widespread SP therapies worldwide, manipulative therapy (MT) is still a pillar because it is a cheap, safe, and easy-to-access approach when compared to other pain therapies [7]. The World Health Organization (WHO) defines spinal MT as “all the procedures where the hands or mechanical devices are used to mobilize, adjust, manipulate, apply traction, massage, stimulate, or otherwise influence the spine and paraspinal tissues with the aim of influencing a patient’s health” [8]. In spine mobilization, therapists slowly move the joint within its normal range of movement, while spine manipulations are the set of forced passive mobilizations coded according to Robert Maigne’s principles [9] and they can be categorized into different techniques: nonspecific, long-lever techniques or specific, short-lever, high-velocity, and low-amplitude techniques [8]. Maigne explained new concepts regarding pain of spinal origin, thus outlining distinctions with different approaches that relate SP to poor mobility of vertebral segments. According to his findings, SP is caused by intervertebral dysfunction of the mobile segments, which can induce pain radiating in the dermatome at the same level as the vertebral problem. Moreover, Maigne enhanced the importance of manipulative treatments of the spine [9]; however, it is mandatory to evaluate the side-effects of these treatments. A systematic review conducted by Bronfort et al. reported common benign transient side-effects (e.g., local muscle and joint soreness) and other complications (e.g., neurological deficits due to lumbar disc herniation) that range from relatively uncommon to extremely rare [7].

They are usually performed not only by medical doctors, especially by physiatrists, but also by other healthcare professionals, such as physiotherapists, practitioners in osteopathy, and chiropractors, depending on the country and the related organization of the healthcare system [10].

Manipulations traditionally guarantee good clinical efficacy in SP relief but they are also a therapy for which it is extremely complex to make results clear and demonstrable. In the literature, traditionally, all these outcomes are about pain (often directly reported from patients), functionality, and psychological dimension outcomes. This limit lies first and foremost in the difficulty of creating order and clarity within the outcome measures, and it causes a gap in the available scientific literature. It is mandatory to make the evaluation tools homogeneous in order to validate the effectiveness of the results of spine manipulations. Thus, the aim of this comprehensive review is to summarize all the evaluation instruments used to assess the effects of spine manipulations, highlighting the limitations and advantages of traditional rating scales and innovative measurement tools.

## 2. Materials and Methods

The literature extensively explores the applications of manual therapy but the effectiveness of validated tools is still debated. So, a comprehensive review was conducted on studies exploring the available evaluation tools used to assess the efficacy of spine manipulation. The manipulation techniques are different and more or less scientifically validated; they are also implemented by different professional figures with different results. What they have in common include the indications and efficacy of pain and functionality in idiopathic spinal pain in the absence of serious pathology with surgical or neurosurgical on-the-spine indications or in the absence of serious underlying oncological pathologies or severe osteoporosis.

**Figure 1:**
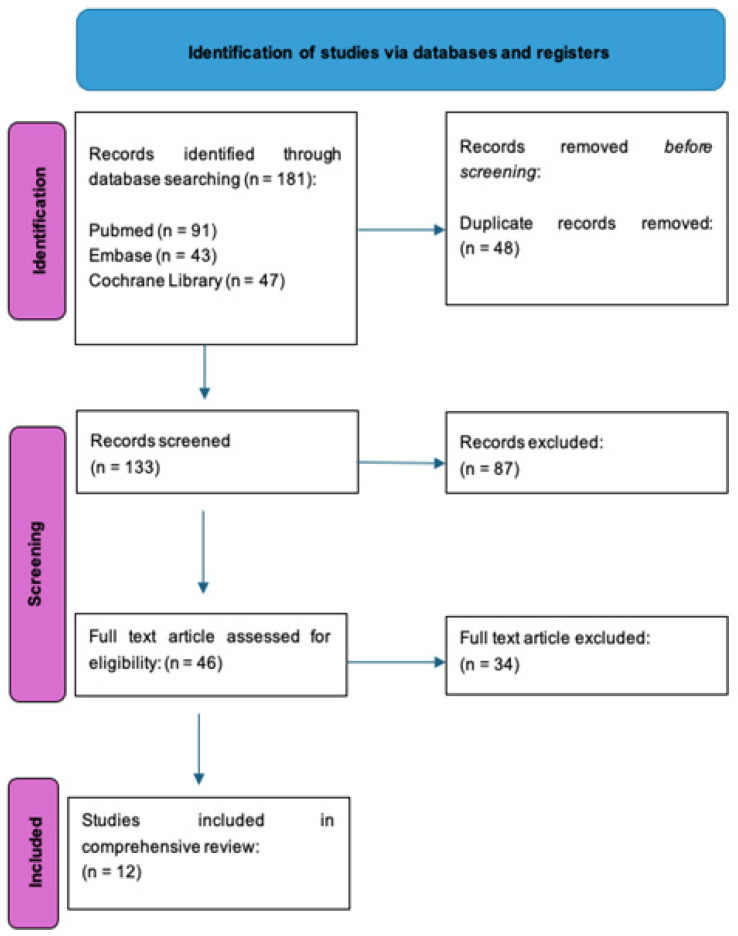
Flow chart of the selected studies.

Three independent authors carried out the article search. The electronic search engines used were PubMed (https://pubmed.ncbi.nlm.nih.gov, accessed on 16 February 2024), Cochrane (https://www.cochranelibrary.com, accessed on 16 February 2024), and Embase (https://www.embase.com accessed on 16 February 2024). The used keywords were “manual medicine“, “spine manipulation”, “assessments”, and “outcome measures”. Several synonyms were searched for each keyword (i.e., manipulation, low back manipulation, neck manipulation, manual treatments, manual therapy, evaluation tools, and efficacy measures). Then, the following filters were activated: test availability: full text; species: humans; languages: English; and period: from 2014 to 2024. The references of the articles were manually examined to find the more relevant publications. Once the potential articles were gathered, they were further filtered based on specific criteria for inclusion or exclusion. The inclusion criteria were the following: all the recent articles (2014–2024) related to manual medicine for spine pain and particularly related to assessment tools (including clinical trials, randomized trials, and study protocol) independently from their level of evidence; online full text available; and papers published in the English language. The exclusion criteria were the following: systematic review, overview, or meta-analysis; duplicated records; and articles published before 2013 or articles about manipulative therapy for other diseases treatment (Figure 1). Only articles published in the last ten years were included as they contain all the most recent SP treatment outcome measures as well as traditional outcome measures that are still routinely used in clinical practice and have therefore not fallen into disuse.

Three investigators (L.D.A, F.Q., and G.F.) separately assessed each title, abstract, and full-text article for eligible studies. Disagreements were resolved by consensus by asking three other experienced investigators (C.M., A.B., and C.S.). The data extracted from each study included the study sample and design, outcome measures, and results. The outcomes of interest were all the measure tools and scales used in manual therapy for treating SP, as follows: (1) self-reported pain measures; (2) self-reported functional measures; (3) objective outcome; and (4) psychological outcome measures. 

As a comprehensive review, statistical analysis and risk of bias assessment were not conducted.

## 3. Results

Based on the aforementioned inclusion and exclusion criteria, a thorough examination of the search results yielded 12 articles, which could be classified into two primary categories: 10 randomized controlled trials and 2 study protocols.

Below is a comprehensive summary including the key points extracted from the selected studies (Table 1).

## 4. Discussion

An analysis of the data gathered from our research revealed that the selected studies consider three categories of outcomes: self-reported outcome measures or patient-reported outcome measures (PROMs), objective outcomes, and psychological outcomes. Specifically, we distinguished PROMs into two categories: pain PROMs and functional PROMs, which were used in several studies as the main outcomes.

### 4.1. Patient-Reported Outcome Measures

Self-reported outcome measures, even defined PROMs, are commonly used in clinical trials to assess the effectiveness of new treatments considering the importance of the patient perspectives during the evaluation and validation of a therapy [23]. Patient involvement guarantees that the considered outcomes are closely relevant to the circumstances of the target population. This kind of measure is developed to evaluate invisible health factors such as pain, health-related quality of life, physical function, or psychological aspects. Certainly, it is mandatory to also consider that individual factors are relevant in self-reported measures and that core concepts could be experienced in different ways across different populations. Thus, it is recommended that the development of content validity guidelines and the use of generic measures provide a foundation for PROMs [24].

#### 4.1.1. Pain PROMs

The perception of pain has conventionally been considered as a subjective assessment; otherwise, the measurement of pain is an essential outcome for clinical studies.

Several of the selected studies considered pain as the main outcome of the research [11,12,13,18,19,21] and a common type of measurement was the visual analogue scale (VAS), which is the most frequently used self-reported measurement for pain intensity in LBP trials [25].

It consists of a 10 cm long vertical line with two endpoints labeled as “no pain” and “maximal pain” without intermediate markers. The patient is asked to mark the level of pain intensity in this line considered as a continuum between the two extremes of pain perception [13] and the score is measured considering the distance between the “no pain” endpoint and the patient’s mark [26].

Previous studies supported the validity and reliability of this measure across many populations [27,28,29,30], although a study reported drawbacks related to practical situations about the clinician’s measurement of the patient’s line and the difficulty of measurement in case of length changes in the line [26]. VAS was considered a main measurement of pain for different studies that considered patients with NP as the sample. In a recent randomized controlled trial (RCT), VAS was used with the aim of investigating the effectiveness of manual therapy in the cervical spine during a 12-week treatment in patients with non-specific chronic NP [18]. Pain intensity was one of the two primary outcome measures and it was used before, after the treatment, and during a follow-up conducted four months after the beginning of the trial. Gonzalez Rueda et al. used the VAS in order to assess the effects on pain intensity of two different specific manual techniques in patients with chronic mechanical NP [19]. Even in the RCT conducted by Siddiqui et al., the authors chose to use VAS for the evaluation and monitoring of mechanical NP, although one of the limitations reported by this research was that the change in pain intensity was limited to subjective findings of the VAS only [13]. In an RCT study protocol conducted by Simson et al., VAS was included in a battery of self-reported online questionnaires to evaluate the influence of intervention on an individual’s perception of treatment in a group of adults with chronic non-specific LBP [21].

VAS is not the only scale used to assess the pain intensity perceived by patients. In fact, many researchers have attempted to overcome the limitations of the VAS as a purely visual scale. Another common measure of pain intensity is the Numerical Rating Scale (NRS). It is a valid and reliable [26,31,32] scale that is made up of 11 points from 0 to 10, where 0 represents “no pain” and 10 represents “maximal pain”. When patients report pain intensity, they have to select the single number that best matches their perception. In the selected studies, NRS was considered in order to evaluate the efficacy of manipulative treatment [12,16] as additional information about the sample population or as a measure for eligibility criteria [16,22]. Thomas et al. used the same scale, even if in their study they called it the Numerical Pain Rating Scale (NPRS), to monitor changes in pain during the treatment [11]. A reduction in more than 2 points in this scale between the baseline score and the score after the treatment was considered a clinically meaningful important difference based on previous studies [33]. Another pilot RCT used NPRS for the measurement of average 24-hour pain and presented pain index (PPI 0 to 10) as the secondary outcome with the aim to investigate the effectiveness of two different approaches of mobilization in patients with NP [15].

Bialosky et al. used the 101-point numeric rating scale (NRS-101) with the aim of evaluating LBP after two weeks of spinal MT. This scale was also used in the same study for the suprathreshold heat response assessment [16]. With the NRS-101, patients are asked to rate their pain extent on a numerical scale from 0 to 100 where 0 represents the minimum extreme (e.g., “no pain”) and 100 represents the maximal extreme (e.g., “pain as bad as it could be”). The NRS-101 could be conducted either in written or verbal form and this is a practical advantage related to other types of measurements such as VAS, which could be administered only in a written form. Moreover, NRS-101 has 101 potential responses that make it more acceptable to clinicians and researchers [25].

Another type of scale is the 11-box scale used in the study protocol designed by Vihstadt and colleagues [22]. The system of two extremes is similar to the previously described scales but, in this case, there are 11 boxes numbered from 0 to 10 and patients are asked to mark the box that better represents their pain intensity perception, considering 10 as the worst pain that could be perceived. The aim of this scale in the study was to evaluate pain as a secondary outcome at each detection time. Nevertheless, this scale does not differ significantly from VAS and NRS as it is limited to a quantification of pain that does not take into account all the biopsychosocial aspects of pain.

As a consequence, in the field of pain PROMs, there are other useful tools that focus attention on pain from a multidimensional point of view. The McGill Pain Questionnaire (MPQ) is an interviewer-administered battery of questions related to sensory, affective, and evaluative aspects of pain in adults with chronic pain. Therefore, it is useful to evaluate the efficacy of pain interventions and to identify various qualitative nuances of pain. Bakken et al. used MPQ for the assessment of the quality of pain after spinal MT and home stretching exercises in patients with persistent or recurrent NP [12]. During this questionnaire, divided into subclasses, patients have to choose the word that best matches their perception of pain.

Previous studies investigated the reliability and validity of this measure: test–retest reliability was considered high to low meanwhile content and construct were considered valid, particularly to evaluate mild pain considering the multidimensional nature of this scale. Although it was translated to represent 26 different languages and cultures, there are limitations related to the words used in this questionnaire. The elaborate and not well-known vocabulary could lead to a failure to understand instructions and consequently to lose significant information [34].

In their RCT, J.M. Pugliese et al. considered even the evaluation of movement-evoked pain during and after functional tests using the 6-Minute Walk Test (6MWT) and the 30 Seconds Chair Stand Tests [20]. Movement-evoked pain is an interesting pain analysis from a different perspective because it could lead to a better understanding of the relationship between pain and movement [35]. A smaller increase in pain implicates a lower pain provocation with activity; nevertheless, in the study design, it is not well described as to how the score could range and how it was administered [20].

Regarding of LBP, subjective complaints are often an integral part of the documentation considered for patient evaluation. In this regard, the Subjective Complaints Questionnaire for low back pain (SCQ-LBQ) is a useful tool for clinical researchers. In this questionnaire nature of symptoms, including both aggravating and easing factors, 24-hour behavior and the history of pain are considered particularly. The test–retest reliability of this measurement has proven to be moderate. However, further studies with a larger sample are needed for full and clear validity [36]. In their study, Simson et al. [21] focused on this questionnaire and several others as well a protocol for an RCT concerning LBP. Another questionnaire used in this study protocol in terms of pain is the Sciatica Frequency and Bothersomeness Index [37], which was used to ascertain further symptoms related to back pain considering even leg pain.

González Rueda et al. [19] used the Headache Impact Test (HIT-6) in order to evaluate the effectiveness of a manual therapy for NP. HIT-6 is a questionnaire with good test–retest reliability [36], which consists of questions about the intensity of pain, social functioning, vitality, cognitive function, and psychological disorder [38]. This survey aims to quantify the impact of headaches on sufferers’ lives considering a total score ranging from 36 to 78 and related to the frequency of headaches in several situations [39].

Furthermore, spine MT leads to changes in pain sensitivity; the research of Bialosky et al. focused on this aspect, assessing pain sensitivity, specifically mechanical and thermal pain sensitivity [16]. For mechanical pain sensitivity, the suprathreshold was assessed using a pressure algometer and a 100 mm mechanical visual analog scale (MVAS). In terms of thermal pain sensitivity, it was assessed using a 101-point NRS after heat stimulation [16]. In general, the use of this method of investigating pain sensitivity is still very limited in the literature relating to MT, so it would be appropriate to evaluate its replicability in future studies based on large populations.

Pain measurement remains an open challenge for healthcare professionals. Starting from the traditional tools listed above, which allow us to effectively investigate various nuances of patients’ perception of SP, the scientific community is called upon to carry out new research aimed at experimenting with new methods to define it, in particular using technologies that study movement and major musculoskeletal functions affected by pain.

#### 4.1.2. Functional PROMs

Similarly to what was explained before about self-reported outcome measures for pain, functional status and health-related quality of life are outcomes that are commonly collected directly from what patients report using standardized questionnaires [22].

With regard to LBP, various functional self-reported scales have been developed and utilized in clinical trials to evaluate the effectiveness of MT.

The Roland-Morris Disability Questionnaire (RDQ) and the Oswestry Low Back Pain Disability Questionnaire are two of the most used scales for the assessment of disability in people with LBP [40].

Despite the fame of the RDQ, it was used in only one of the selected studies of this research [11]. Since the first release in 1983, various revisions of this scale have been proposed regarding the items’ number or content. Regardless, the original version is still the recommended one [41].

This questionnaire is composed of a list of 24 items regarding several activities and functions of daily living such as sleeping, mobility, and housework. If the item matches the patient’s situation, they have to check the related box or leave it blank if the sentence does not state their feeling. The final score is related to the number of items checked and so it could range from 0 to 24 and then, respectively, from absence of disability to maximal disability. Previous studies affirmed the good internal consistency of this measurement even though they considered higher reliability for shorter times than for longer intervals [42]. This questionnaire is simple and easy to understand for patients and therapists but it considers only 24 items about LBP despite the higher number of possible different activities that could be related to this issue. Moreover, many psychosocial factors such as appetite and irritability are not included in this tool [41].

The Oswestry Low Back Pain Disability Questionnaire was developed first in 1980 to assess pain-related disability regarding LBP [43]. It is made up of 10 items regarding various aspects of function. For each item, there are six possible scenarios ranging from the best to the worst and related to a score from 0 to 5. At the end of the questionnaire, the total amount of scores of the single items determines the Oswestry Disability Index (ODI) [44]. Several researchers used the ODI in their RCT considering its good construct validity and reliability and responsiveness over short intervals [21,45,46]. Vihstadt et al. considered back disability as the main outcome of their study protocol and consequently, the ODI was the measurement used to assess the effectiveness of the treatment, composed of spinal MT and supervised rehabilitative exercise, in a population of adults aged more than 65 years [22]. A revised version of this questionnaire was used to implement the assessment measures of the study protocol for an RCT including patients with chronic and non-specific LBP lasting more than 3 months [21]. As performed by Bialosky et al., the total score is usually multiplied by 2 and then expressed as a percentage where higher scores indicate greater disability [16]. Furthermore, the Oswestry Low Back Pain Disability Questionnaire was used as the basis for the development of another measurement related to LBP, namely the North American Spine Society (NASS) outcome–assessment instrument. From the NASS questionnaire, Bialosky et al. used two questions aimed at evaluating patients’ satisfaction considered as a secondary outcome of their study [16]; this could represent an interesting new key to using this scale.

With regard to neck disability, many of the selected studies used the Neck Disability Index (NDI) in their research [12,13,14,15,18,19,22].

The NDI is a measure that is reliable, valid, and strongly consistent in terms of responsiveness concerning patients with mechanical NP [47]. It is a questionnaire, derived from ODI, that is made up of 10 items regarding not only pain but also several factors related to NP and activity of daily living.

Each item has six possible answers representing different progressive levels of disability where 0 is the lower level and 5 is the highest level. The score of each question is summed at the end of the questionnaire and it reflects the level of disability ranging from no disability to complete disability with mild, moderate, and severe as intermediate levels of disability [13]. Another measurement that has been investigated in order to find a relationship with NDI is the EuroQol 5 dimensions questionnaire (EQ-5D), which is a measurement related to quality of life [48]. This valid measurement [49] has been used in the studies of Vihstadt et al. [22] and Bakken et al. [12] considering the usefulness of reflecting the patient’s health status with an index ranging from 0 (death) to 1 (full health).

Another scale largely used in clinical research and especially in spine studies [50,51,52,53,54] is the Global Rating Of Change (GROC) scale. It could be also known by different titles such as the Global Perceived Effect Scales, Patient Global Impression of Change, Transition Ratings, and Global Scale [55]. The scope of this scale is to quantify the changes in a patient’s health status in terms of improvement or deterioration consequent to the clinical intervention. The GROC scale is conducted using a scale with positive and negative numbers where 0 is the midpoint that stands for no change, positive numbers represent a growing enhancement, and, conversely, negative numbers represent a decline [56]. Several variations in this measurement could be found in the scientific literature, varying from 7-point to 11-point and 15-point scales. Lagoutaris et al. [15] used an 11-point scale ranging from −5 to 5 in order to evaluate patients’ rating of clinical change in their pilot RCT related to cervical spine mobilization for NP. Simson et al. used the 7-point version of this scale to assess participants’ overall perception of change since study commencement in their study protocol for an RCT [21]. Differently, a 15-point scale was considered as one of the measurements for the study protocol for an RCT designed by González Rueda et al. [19]. The validity of the GROC scale is high and it has strong and significant correlations with RDQ and the Oswestry Low Back Pain Disability Questionnaire. The simplicity of this scale accounts for its widespread use in clinical practice. Nevertheless, it is necessary to take into consideration the weaknesses of the GROC scale that have emerged in other research in relation to patients’ ability to remember their previous state of health [52]. The GROC scale and related variations are not the only measurements regarding the patient’s perception of improvement in his health status. In fact, Vihstadt et al. used a single nine-point ordinal scale to evaluate the improvement perceived by the patient after starting treatment for both back and neck problems [22]. Specifically, the improvement or the reduction in the health status was designed to be assessed in every follow-up starting from the fourth week [22]; in this sense, this scale has proven reliable.

With regard to LBP, the Quebec Back Pain Disability Scale (QBPDS) is a common measurement used to assess physical disability caused by this issue [57]. This 20-item self-report questionnaire has good validity, high internal consistency using the original numerical 11-point scale, and good reproducibility, which makes the use of QBPDS in clinical practice widespread [44]. In fact, it has been included in the trial of Pugliese et al. as a primary outcome measure for physical function in a population of patients with LBP [20]. The items composing the questionnaire refer to activities of daily living that patients may perform with difficulty; they are divided into six domains (i.e., bed/rest, sitting/standing, ambulation, movement, bending/stooping, and handling of objects) [58]. Patients are asked to answer the questionnaire considering the level of perceived difficulty in order to perform the answered activity on the current day. The difficulty value ranges from 0 (“not difficult at all”) to 5 (“unable to do”) and at the end, a total amount of every item score is used to assess the level of disability. So, this scale is potentially very useful in rehabilitation, since it allows us to evaluate the functional capabilities of patients in relation to the musculoskeletal disease that limits them.

Further questionnaires used in one of the selected studies were the Low Back Activity Confidence Scale (LOBACS) and the Hip Disability Osteoarthritis Outcome Score (HOOS) [18]. The first one is a 15-item questionnaire used to assess an individual’s self-efficacy beliefs about performance in back-relevant tasks such as carrying, pushing, exercising, and similar activities [59]. Meanwhile, the HOOS is a questionnaire composed of 40 items divided into 5 domains (pain, symptoms, activities of daily living, sport and recreation function, and hip-related quality of life) and addresses patients with hip issues (e.g., hip osteoarthritis) in order to assess symptoms and functional limitations related to the hip [60]. Given the close biomechanical correlation between the hip and the lumbosacral spine [61], this scale can be very useful for discriminating the origin of pain and, above all, its impact on patients’ motor function. In terms of self-efficacy, a further valid and reliable measurement is the Pain Self-Efficacy Questionnaire, which was used by Vihstadt et al. in their research on older adults with back and neck disability [22]. While completing this questionnaire, patients declare their level of confidence (using values from 0 to 6) when performing several activities in the presence of chronic pain [62], thus configuring the dysfunctional dimension of pain.

Regarding PROMS, the Patient-Reported Outcomes Measurement Information System (PROMIS) is another questionnaire concerning health-related quality of life. The 29 items of this questionnaire are divided into eight domains related to several individual aspects such as physical function, anxiety, depression, fatigue, sleep disturbance, ability to participate in social roles and activities, pain interference, and pain intensity. It is an efficient, flexible, and precise measurement commonly used in the field of patient-reported outcomes and, specifically, in relation to patients with chronic back and hip pain [20].

Evaluating the effectiveness of a treatment in terms of functionality is a highly useful aspect to consider. Most of the selected studies used questionnaires and scales aimed at emphasizing the quality of life and disability changes related to SP, especially in the activities of daily living. However, it seems necessary to include further items that could better target the fundamental aspects that researchers aim to explore with these outcome measures, to make them more objective and replicable.

### 4.2. Objective Outcome Measures

To better understand the manual therapy efficacy, objective and reproducible methods are essential for the scientific validity of a clinical trial.

Pain involves a “biomechanical load” that affects movement and function [63]. If pain can be hardly investigated using measurement tools that are not patient-reported, on the contrary, mobility, strength, functional improvement, endurance, and performance are aspects that can be easily assessed with some popular and objective tests. The majority of measurement methods in the literature involve the use of some simple or more sophisticated evaluation tools.

In particular, patients who suffer from NP often experience a reduction in neck mobility due to pain and muscular contractures [64]. One of the most used and feasible evaluations is active cervical mobility and the passive cervical range of motion (ROM).

To show an increase in cervical mobility, several studies performed assessments measuring active cervical ROM, evaluated in all planes for global cervical mobility and in the sagittal plane for the upper cervical spine. For the active cervical ROM testing, patients were asked to sit with a straight neck and move their head as far as possible in a free-from-pain range [65].

Gonzalez Rueda et al., both in the 2020 study and in the 2017 study protocol [14,19], included this active cervical ROM to objectify the efficacy of the manual approach to the suboccipital region in patients with chronic mechanical NP and rotation deficit in the upper cervical spine. Moreover, to evaluate the passive upper cervical spine ROM, they included the “Flexion–rotation test”. It was performed with patients in the supine position while the examiner passively took the subject’s cervical spine to its maximum flexion, rotating the head to the right and left side. The movement stops when the subject presents symptoms or the evaluator reaches the end of the ROM.

It is essential for clinicians to have reliable and valid measurement instruments in order to objectively monitor disease progression, outcomes, and mobility impairments.

Siddiqui et al. [13] evaluated cervical ROM in passive flexion and extension using a goniometer and a digital inclinometer, instruments with a good reliability coefficient [66]. These tools are simple and handled devices commonly used in clinical practice. The ROM evaluation occurred for cervical flexion and extension with the goniometer in the center of the ear; for cervical lateral flexion on both sides with the goniometer placed on C7 spinous process; and for cervical rotation left and right placing it on the top of the head.

Lagoutaris et al. [15] used a cervical inclinometer for the range of motion measurements (CROM) device, which is easily available and widely used in physical therapy. The handheld goniometer is an easily accessible and convenient clinical tool but is limited by an inability to record complex movements with greater than one center of rotation.

In their study about Manual Therapy in the Cervical Spine and Diaphragm, Tatsios et al. [18] also evaluated cervical ROM in neck flexion–extension, left–right side flexion, and left–right rotation, with the help of a smartphone-based application downloaded to a smartphone, in parallel with a sensor-based commercially available device. Moreover, with the help of another smartphone-based application, they also measured the Craniovertebral Angle (CVA), calculated with patients in the forwarded head position and relaxed sitting position, through lateral photographs. Although it is difficult to establish the precision of these digital instruments, they certainly represent an interesting prospect as they are an example of the practical application of new technologies to measurements in the musculoskeletal field.

In their multisite randomized trial, Pugliese et al. [20] used several functional outcomes to compare a hip-focused LBP treatment and a spine-focused LBP treatment. They evaluated gait speed with the 10 m walking test, with participants walking along a linear pathway as quickly as possible, where a higher gait speed represents a better outcome. Then, they evaluated the functional mobility using the 6-Minute Walk Test (6MWT), with patients walking around a predetermined course trying to cover as much ground as possible in 6 min, and the 30 Seconds Chair Stand Tests, which entailed asking subjects to perform as many sits to stand as possible in 30 sec with their arms folded across their chest. Regarding the strength of the data, they asked participants to perform hip strength exercises (abduction, extension, internal and external rotation, and flexion) and they used a hand-held dynamometer, normalized to body weight, to assess the muscle capacity.

Carpino G. et al. [17] used a totally different and more sophisticated method to determine whether manual therapy affects functional and biomechanical performance during a sit-to-stand (STS) task in a population with LBP. They recorded data using an optoelectronic motion capture system while patients performed an STS task before and after manipulations. Pelvis and thorax kinematic data were used to derive the variation in thelumbar angle in the sagittal plane for each STS exercise. The difference between the maximum and minimum lumbar angles during the exercise determined the sagittal ROM, collected as a biomechanical outcome. On the other side, the time to complete each exercise was used as a functional measure of performance.

To do that, they used eight cameras to monitor the thorax and pelvis kinematics during the task performance in three dimensions, while individual reflective markers were applied overlying anatomical landmarks on the pelvis and thorax bilaterally over the acromion processes, the iliac crests, anterior superior iliac spines, and the posterior superior iliac spines. This complex measurement system seems to be able to guarantee extremely precise and reliable data but it could be very expensive, difficult to use, and, therefore, poorly widespread.

Vihstadt et al. [22] used a hand-held hydraulic dynamometer to assess the hand grip strength. It is a traditional instrument that is able to evaluate strength well, whose changes can be indirectly determined by pain. These authors also carried out the short physical performance battery (SPPB), used as a predictor of future disability in healthy older adults over the age of 70; it comprises three tests, the gait speed, standing balance, and chair rising [67], and it could be a good tool to evaluate how pain affects an individual’s motor performances.

Simson et al. [21] included the use of MRI to evaluate if their exercise program could positively affect intervertebral disc condition, spine function, and muscle size and quality. This is an extraordinary step forward in the objective evaluation of MT results. The authors carried out a series of scans for each patient to assess the average lumbar spine, the intervertebral disc volume and height, the vertebral body fat content and cortex aspect, the trunk muscle size, the lumbar muscle fat content, and the water diffusion rates of intervertebral disc.

As a secondary outcome, they further referred to the use of technology. To better understand the MT impact on total body composition and bone mineral density, they used an iDXA scanner and a lumbar spine scan, collecting data about the total body lean mass, fat mass, fat percentage, and lumbar bone density.

These measurements can attest extremely precisely to the changes caused by spinal manipulations and they undoubtedly represent the most correct way to follow in order to translate the reduction in pain into objective data from an anatomical point of view.

Moreover, Simson et al. [21] planned to include, among the outcomes, participants’ response at transcranial magnetic stimulation (TMS).

TMS is used both to measure excitatory and inhibitory responses in the connections between the cortex and muscle involved in back pain and to develop a cortical map of the back muscles.

This is a recently developed increasingly popular noninvasive brain stimulation method used in different fields as a treatment or evaluation tool thanks to its effects on the nervous response. Although its exact mechanism of action is still not clear, current evidence points toward its role in causing long-term inhibition and the excitation of neurons in certain brain areas. Despite the fact that evidence steadily grows in favor of TMS as a therapeutic and evaluation tool, there is still a need to develop standardized protocols for its correct use [68,69].

In conclusion, new technologies and digital tools represent a potential turning point in evaluating the effectiveness of manipulations for the treatment of SP but larger studies and cheaper and easier-to-use technologies are indispensable to achieve this goal.

### 4.3. Psychological Outcome Measures

Psychological measures are known to influence treatment efficacy in terms of pain perception, functional outcomes, and adherence to therapy.

Several studies were used as the secondary outcome to some psychological questionnaires, with the aim of measuring the impact of psychological factors on pain.

An essential aspect of pain perception is related to cognitive factors. Particularly, the scientific literature focused on catastrophizing, which is generally defined as an overstated and negative mental set related to a painful experience [70]. This could affect pain perception and the patient’s ability to cope effectively with pain. Sullivan et al. developed the Pain Catastrophizing Scale (PCS) to assess catastrophizing in clinical and nonclinical populations [71]. The PCS is made up of 13 items divided into three subscales which focus on three different constructs: rumination, magnification, and helplessness. This valid and reliable scale [72] was implemented in two of the selected studies. Specifically, Pugliese et al. [20] aimed to evaluate how manual therapy and strengthening of the hip could affect performance and disability in a population with chronic LBP; they used PCS in the domain of pain perception to better assess their manipulations’ effects. Similarly, Bialosky et al. [16] included this scale in a battery of psychological questionnaires. They also administered the Fear and Avoidance Belief Questionnaire (FABQ), which focused specifically on patients’ beliefs about how physical activity and work affected their LBP and the Tampa Scale of Kinesiophobia (TSK) [73], one of the most used outcome measures for assessing pain-related fear in back pain patients. These choices are considered as very interesting as they shed light on the great problem of kinesiophobia, which can significantly modify the motor outcomes of patients suffering from SP. Also, it is well known that they affect the estimate of pain reported by the patients themselves.

Frequently, musculoskeletal research includes anxiety and depression assessments.

Pugliese et al. [20] used the Patient Health Questionnaire-9 (PHQ-9), a questionnaire with nine items related to depressive symptoms, to diagnose, monitor, and determine the severity of depression. PHQ-9 consists of two questions: the first one investigates the presence in the previous two weeks of the nine symptoms of depression according to the Diagnostic and Statistical Manual of Mental Disorders (DSM). The second one assesses the functional impairment caused by depression in patients’ lives.

A big part of the study protocol by Simson et al. [21] is dedicated to the psychological dimension of pain, with an eye on quality of life, sleep, physical performance, and work activities [73,74,75,76,77,78,79]. They included a battery test like the Center for Epidemiological Studies-Depression Scale (CES-D) 10 [80], a scale that considers a self-report measure of depression and the Positive and Negative Affect Schedule (PANAS) [81], a tool to evaluate the affective state of patients. Finally, Vihstadt et al. [22] also included the Geriatric Depression Scale [82], a scale designed to screen depression and cognitive impairment incidence in elders.

The efforts of all the mentioned above authors were aimed at highlighting the impact of the psychological state of patients on pain, thus highlighting how all treatments, including manipulations, are limited by this aspect and how it is important that healthcare professionals consider it when proposing and carrying out such therapies.

### 4.4. Limitation of the Study

This study is not free from limitations. First of all, we decided to not deepen and extensively discuss all the outcomes mentioned in the selected articles; we chose only the outcomes directly or indirectly related to pain and functionality, the two main aspects related to spinal manipulation treatments.

Moreover, in the literature, there is no consensus about the spine manipulation definition. In fact, several manipulative techniques are applied around the world with different theoretical–practical approaches and even different denominations. For this reason, we can not exclude the chance that some studies on this issue have been excluded; but, we highlighted all the outcome measures analyzed in the selected studies, which represent the most important contribution in this matter. Furthermore, this investigation considered various types of spinal issues with different related backgrounds. Thus, it is important to take into account this consideration when comparing different outcomes.

On the other hand, this study offers a detailed and thorough examination of the most recent scientific evidence regarding spine manipulation effectiveness, emphasizing the limitations and advantages of traditional rating scales and innovative tools and highlighting the lack of homogeneity of the outcome measures used in the various studies. As a consequence, the most challenging aspect of making results clear and demonstrable is the difficulty in creating order within the numerous outcome measures.

To our knowledge, this is the first study that tries to fill this gap and focuses on the evaluation instruments used to assess the effects of spine manipulations.

However, further studies are needed to focus on a more targeted exploration of each outcome measure, especially using systematic reviews and meta-analyses, with the aim of make the MT effects increasingly objective and replicable.

## 5. Conclusions

This comprehensive review primarily provided an in-depth exploration of the outcome measures used to assess the efficacy of MT for SP.

The perception of pain by a patient has conventionally been considered as a subjective assessment, so it is very difficult to study and measure pain, especially in order to evaluate the effectiveness of a manual therapy such as manipulations.

The traditional self-reported scales for assessing pain are still valid tools but it seems mandatory to include them in the evaluation of the MT efficacy for SP as well as scales and instruments based on patients’ psychological status and on the musculoskeletal structure’s functions, since strength, joint ROM, and motor ability are strictly linked with pain. In this sense, new technologies and digital approaches, such as MRI, sEMG, accelerometer, and mobile applications, could represent a great help to test the manipulations’ effectiveness and to verify the results in an increasingly precise and objective way. Nevertheless, these technologies still need to be better developed to make them more affordable, accessible, and easy to use, just as new studies are needed to validate their effectiveness on larger patient populations. Moreover, it is essential to find increasingly objective evaluation scales to support the effectiveness of MT and consolidate their importance in international guidelines for the non-pharmacological management of SP.

## Figures and Tables

**Table 1:** Selected articles summary.

Title	Authors and Publication Year	Study Sample and Design	Outcomes of the Study	Results
Effect of Spinal Manipulative and Mobilization Therapies in Young Adults With Mild to Moderate Chronic Low Back Pain: A Randomized Clinical Trial.	Thomas J.S. et al., 2020 [11]	RCT—162 patients with chronic LBP qualified for randomization to 1 of 3 treatment groups. Participants received 6 treatment sessions of (1) spinal manipulation, (2) spinal mobilization, or (3) sham cold laser therapy (placebo) during a 3-weeks period.	NPRS, RDQ	At the primary end point, there was no significant difference for pain and self-reported disability scores between spinal manipulation and spinal mobilization, and between spinal manipulation and placebo.
The effect of two weeks of spinal manipulative therapy and home stretching exercises on pain and disability in patients with persistent or recurrent neck pain; a randomized controlled trial.	Bakken A.G. et al., 2021 [12]	RCT—131 adult subjects with recurrent neck pain were randomized in two groups. Both groups received 4 treatments for 2 weeks, spinal manipulative therapies and home stretching exercises compared to home stretching exercises alone.	NRS-11, MPQ, EQ-5D, NDI	There were no statistically significant differences between the groups for any of the outcome measures.
Effects of autogenic and reciprocal inhibition techniques with conventional therapy in mechanical neck pain—a randomized control trial	Siddiqui M. et al., 2022 [13]	RCT—80 patients randomized in two groups. Group 1 received autogenic inhibition with conventional treatment and group 2 received. Reciprocal inhibition with conventional physiotherapy treatment.	VAS, Goniometer, NDI	There was a more significant improvement in pain, disability, neck ROMs in flexion, extension, right and left lateral flexion, and right and left rotation in the group 1 than in the group 2 after the last session.
Does Upper Cervical Manual Therapy Provide Additional Benefit in Disability and Mobility over a Physiotherapy Primary Care Program for Chronic Cervicalgia? A Randomized Controlled Trial.	González-Rueda V. et al., 2020 [14]	RCT—78 patients with chronic neck pain and restricted upper cervical rotation were randomized into three groups: the upper cervical translatoric mobilization group, inhibitory suboccipital technique group, and control group.	NDI, active cervical mobility, flexion-rotation test	The addition of manual therapy to a conventional physical therapy protocol for the upper cervical spine increased the flexion-rotation test in the short and mid-term in patients with chronic neck pain. No changes were found in the NDI or in the global active cervical ROM.
Approaches to cervical spine mobilization for neck pain: a pilot randomized controlled trial.	Lagoutaris C et al., 2020 [15]	Pilot RCT—20 adults with mechanical NP, randomly allocated to either pragmatic or prescriptive mobilization intervention groups.	NDI, NPRS, CROM, Global Perceived Effect	The primary outcome of change in disability scores at 48 h follow-up was not significantly different between the pragmatic and prescriptive group. Global perceived effect of treatment was significantly higher in the pragmatic group. Secondary outcomes of pain and ROM were not significantly different between groups.
Spinal manipulative therapy-specific changes in pain sensitivity in individuals with low back pain (NCT01168999)	Bialosky J.E. et al., 2014 [16]	RCT—110 participants with LBP were randomly assigned to receive Spinal Manipulative Therapy (SMT), placebo SMT, or no intervention. Participants receiving the SMT and placebo SMT received their assigned intervention 6 times over 2 weeks.	NRS, ODI, NASS Lumbar Spine Outcome Assessment, MVAS, FABQ, TSK, PCS	A reduction in pain sensitivity was greater in response to SMT than in response to the expectation of receiving an SMT.
Does manual therapy affect functional and biomechanical outcomes of a sit-to-stand task in a population with low back pain? A preliminary analysis	Carpino G. et al., 2020 [17]	RCT—40 participants suffering from LBP underwent Manual therapy (MT) treatment consisting of two high-velocity low-amplitude spinal manipulations, two grade IV mobilizations of the lumbar spine and pelvis.	Optoelectronic motion capture system; Pelvis and thorax kinematic data; STS	After MT, lumbar sagittal ROM increased and time to complete the STS test decreased. MT might influence the biomechanical and functional performance of an STS task in a population suffering from LBP.
The Effectiveness of Manual Therapy in the Cervical Spine and Diaphragm, in Combination with Breathing Reeducation Exercises, in Patients with Non-Specific Chronic Neck Pain: Protocol for Development of Outcome Measures and a Randomized Controlled Trial	P.I. Tatsios et al., 2022 [18]	RCT—90 adult volunteers of both genders, aged between 25 and 65 years, and with mechanical chronic NP, were divided in 3 groups: group A underwent cervical manual therapy, diaphragm manual therapy, and breathing education exercise; group B underwent cervical manual therapy with soft tissue therapeutic techniques, plus sham diaphragm MT; group C underwent typical conventional physiotherapy.	NDI, VAS, cervical ROM, CVA, HADS, TSK	Release of the diaphragm, in combination with breathing reeducation, decreased pain and other musculoskeletal-related outcomes, and also improved the body’s ability to achieve homeostasis
Effectiveness of a specific manual approach to the suboccipital region in patients with chronic mechanical neck pain and rotation deficit in the upper cervical spine: study protocol for a randomized controlled trial.	V González Rueda et al., 2017 [19]	RCT—78 participants randomly distributed into three groups. The control group received a protocolized treatment, the mobilization group received the same protocolized treatment and 6 sessions of the translatory dorsal glide mobilization (TDGM) C0-C1 technique, and the pressure group received the same protocolized treatment and 6 sessions of the pressure maintained suboccipital inhibition technique (PMSIT).	VAS, NDI, cervical ROM, HIT-6, GROC scale	An approach including manual treatment to upper cervical dysfunction was the more effective in these patients. The PMSIT technique affected mostly the musculature, while the TDGM technique affected the joint.
The Manual Therapy and Strengthening for the Hip (MASH) Trial: Protocol for a Multisite Randomized Trial of a Subgroup of Older Adults With Chronic Back and Hip Pain.	JM Pugliese et al., 2022 [20]	Study protocol—180 people aged between 60 and 85 years with chronic LBP and hip pain were recruited. They underwent a comprehensive baseline assessment and are randomized into 1 of 2 intervention arms: hip-focused or spine-focused treatment.	QPBDS, 10MWT, PHQ-9, LOBACS, PCS, Movement-evoked pain, 6MWT, 30-Second Chair Stand Test, HOOS, PROMIS-29.	As a protocol, no results were available.
Optimising conservative management of chronic low back pain: study protocol for a randomised controlled trial.	KJ Simson et al., 2020 [21]	Study protocol—Forty participants, 25–45 years old with chronic non-specific LBP were randomized to undergo either motor control and manual therapy (n = 20) or general strength and conditioning (n = 20) exercise treatments for 6 months.	MRI, dual energy X-ray absorptiometry, transcranial magnetic stimulation, (SCQ-LBQ), VAS, Sciatica Frequency and Bothersomeness Index, CES-D 10, PANAS, Work Productivity and Activity Impairment Questionnaire, ODI, PSQI, TSK, EWPS, GROC scale.	As a protocol, no results were available.
Short term treatment versus long term management of neck and back disability in older adults utilizing spinal manipulative therapy and supervised exercise: a parallel-group randomized clinical trial evaluating relative effectiveness and harms	Corrie Vihstadt et al., 2014 [22]	Study protocol: 200 adults ≥ 65 years of age with back and neck disability lasting at least 12 weeks.	ODI version 2.0, NDI, 11-box scale, EQ-5D, single nine-point ordinal scale, Self-Efficacy Questionnaire, TSK, seven-point scale, hand grip strength, SPPB, accelerometry, qualitative interviews, Geriatric Depression Scale	As a protocol, no results were available.

The table explains the details of each selected study. The first and second columns list the title and authors of the study, respectively; the third column describes the sample and study design; the fourth column explains the intervention performed; the fifth column presents the results; and the sixth and final column outlines the study’s limitations. Numerical Pain Rating Scale (NPRS); Roland-Morris Disability Questionnaire (RDQ); 11-point Numeric Rating Scale (NRS-11); McGill Pain Questionnaire (MPQ); EuroQol 5 Dimensions Questionnaire (EQ-5D); Neck Disability Index (NDI); Visual analog scale (VAS); Cervical inclinometer for Range Of Motion (CROM); Oswestry Disability Index (ODI); North American Spine Society (NASS); Mechanical Visual Analog Scale (MVAS); Fear Avoidance Belief Questionnaire (FABQ); Tampa Scale of Kinesiophobia (TSK); Pain Catastrophizing Scale (PCS); Sit To Stand (STS); Craniovertebral Angle (CVA); Hospital and Anxiety Depression Scale (HADS); Range Of Motion (ROM); Headache Impact Test (HIT-6); Global Rating Of Change scale (GROC scale); Quebec Back Pain Disability Scale (QBPDS); 10-Meter Walk Test (10MWT); Patient Health Questionnaire-9 (PHQ-9); Low Back Activity Confidence Scale (LOBACS); 6-Minute Walk Test (6MWT); Hip Disability Osteoarthritis Outcome Score (HOOS); Patient-Reported Outcomes Measurement Information System (PROMIS); Subjective Complaints Questionnaire for low back pain (SCQ-LBQ); Centre for Epidemiologic Studies Short Depression Scale (CES-D 10); Positive and Negative Affect Schedule (PANAS); Pittsburgh Sleep Quality Index Questionnaire (PSQI); Endicott Work Productivity Scale (EWPS); Short Physical Performance Battery (SPPB).
